# Injectable *in Situ* Cross-linked Oxidized Alginate-Gelatin-Based Hydrogels for Cartilage Tissue Engineering

**DOI:** 10.1021/acsbiomaterials.5c01832

**Published:** 2026-02-18

**Authors:** Lisa Schöbel, Juri Artes, Markus Lorke, Aldo R. Boccaccini

**Affiliations:** Institute of Biomaterials, Department of Materials Science and Engineering, Friedrich Alexander-University Erlangen-Nuremberg, 91056 Erlangen, Germany

**Keywords:** alginate, gelatin, injectable hydrogel, *in situ* cross-linking, cartilage tissue engineering

## Abstract

The present study introduces an injectable oxidized alginate-gelatin hydrogel system for cartilage tissue engineering, employing a combination of covalent and noncovalent cross-linking mechanisms. Specifically, the network is formed through Schiff’s Base reactions alongside enzymatic and ionic cross-linking. The hydrogels were investigated regarding their mechanical properties, swelling and degradation behavior, injectability, and cytocompatibility. The results indicated tailorable mechanical properties with an effective modulus ranging from 12 to 20 kPa, depending on the enzymatic cross-linker concentration, while demonstrating suitable injectability required for clinical applications with injection forces in the range of 3–5 N. Moreover, the syringe-mixing approach of *in situ* cross-linked hydrogels showed favorable cell–material interactions with chondrogenic ATDC5 cells.

## Graphical abstract

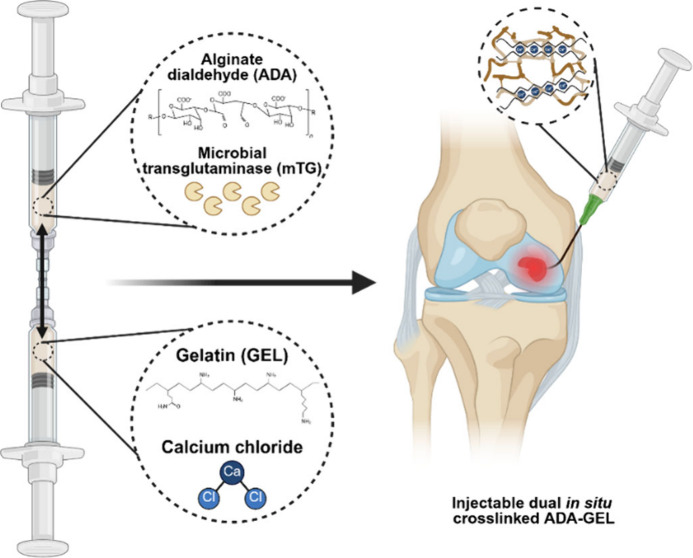

Despite advances in global health, the incidence of osteoarthritis (OA) continues to rise, driven by aging and obesity.^1^ The challenges in treating OA stem from the limited regenerative capacity of articular cartilage (AC) due to the lack of blood vessels and low turnover of chondrocytes.^2^ As current treatments fail to regenerate cartilage long-term, the focus has shifted toward tissue engineering. Injectable natural polymer-based hydrogels represent a promising alternative for cartilage tissue engineering (CTE) due to their biocompatibility, biodegradability, extracellular matrix-mimicking structure, cell encapsulation ability, and suitability for minimally invasive administration, especially in irregularly shaped sites.^1,3,4^ One example of a natural polymer-based hydrogel is alginate dialdehyde-gelatin (ADA-GEL), which has been extensively investigated for various tissue engineering applications over the past years due to its promising properties.^5–7^ It is well-known that a cross-linking step is required to produce hydrogel samples with long-term stability. In the case of ADA-GEL, Distler et al.^6^ proposed a dual post cross-linking approach by immersing hydrogel samples in a cross-linking solution composed of 0.1 M CaCl_2_ and microbial transglutaminase (mTG) to realize the ionic cross-linking of ADA and enzymatic cross-linking of GEL.^6^ However, this post-cross-linking approach is inherently less controllable due to its dependence on diffusion, which can result in a spatially heterogeneous cross-linking. Therefore, *in situ* cross-linking of alginate-gelatin hydrogels represents a promising strategy with various applicable cross-linking methods, e.g., based on Schiff’s Base formation, internal ionic gelation due to the release of ions from inorganic fillers, or enzymatic cross-linkers, such as mTG (see Table S2 for a literature overview). The combination of *in situ* ionic and enzymatic cross-linking for ADA-GEL to produce injectable hydrogels for CTE has not yet been reported. Consequently, we herein investigated an *in situ* cross-linking strategy by following a similar approach to that proposed by Abroug et al.^8^ By incorporating the cross-linking agents directly into the hydrogel precursor solutions, we aimed to produce injectable hydrogels omitting the post cross-linking step while enhancing cross-linking homogeneity and simultaneously reducing the amount of enzymatic cross-linker required. However, this approach rendered conventional beaker-based mixing impractical due to rapid gelation. Consequently, a syringe-based mixing strategy was developed to combine mTG-supplemented ADA with gelatin dissolved in 0.1 M CaCl_2_. With this mixing approach, we envision the use of a double-syringe delivery system similar to that proposed by Ding et al.^9^ The syringe-mixing strategy enabled *in situ* cross-linking via multiple mechanisms, including Schiff-base formation between ADA and gelatin, isopeptide bond formation between adjacent gelatin chains, and ionic cross-linking of ADA (see scheme in Figure 1).

**Figure 1:**
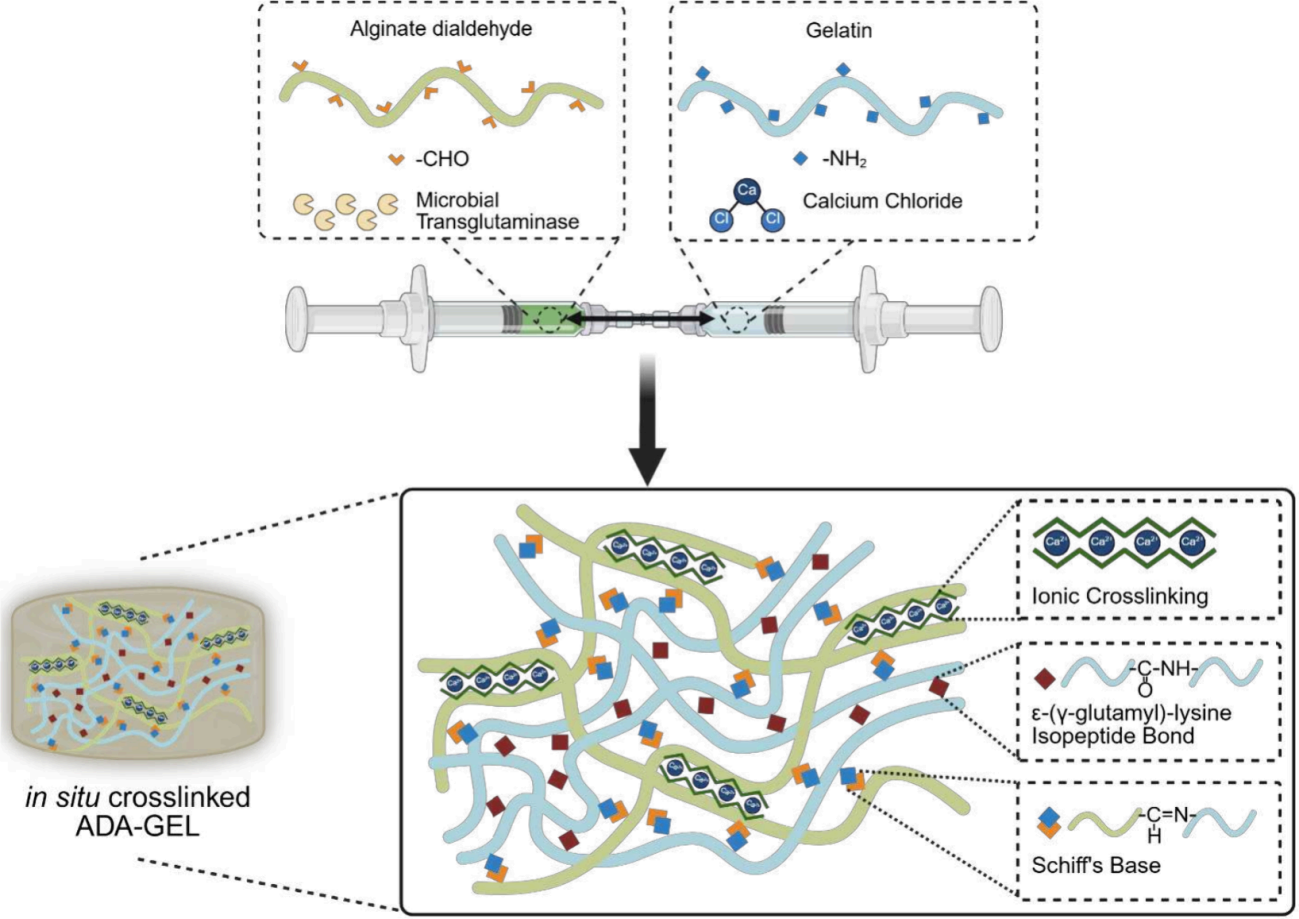
Schematic diagram of the *in situ* cross-linking approach of injectable ADA-GEL.

The impact of the cross-linking strategy and mTG concentration on the long-term stability of hydrogel films under cell culture conditions was investigated by determining the weight change (Figure 2A). It became evident that AG-Ref, AG-5mTG, and AG-10mTG showed distinctive weight loss within the incubation period of 14 days. In contrast, AG-25mTG and AG-50mTG produced with higher mTG concentrations showed increased stability with no observed weight loss within 14 days of incubation. Similarly, the degradation rate determined by solid mass shows a comparable trend (Figure 2B). Taken together, the results suggested that a higher mTG concentration leads to an increased formation of ε-(γ-glutamyl)­lysine bonds between gelatin chains and, thus, an improved resistance to degradation, as reported by Zhao et al.^10^ for mTG-cross-linked collagen films. Therefore, we can tailor the degradation profiles based on the mTG concentration for the *in situ* cross-linking approach. Moreover, AG-25mTG and AG-50mTG were more stable compared to the ADA-GEL reference, while the mTG material consumption was drastically reduced, representing a great advantage of the *in situ* cross-linking strategy. Additionally, the biodegradation of hydrogel films in the presence of collagenase type II was studied (Figure S1), and it was observed that all compositions degraded within 4 h of incubation at 37 °C with slightly slower degradation for increasing mTG concentration.

**Figure 2:**
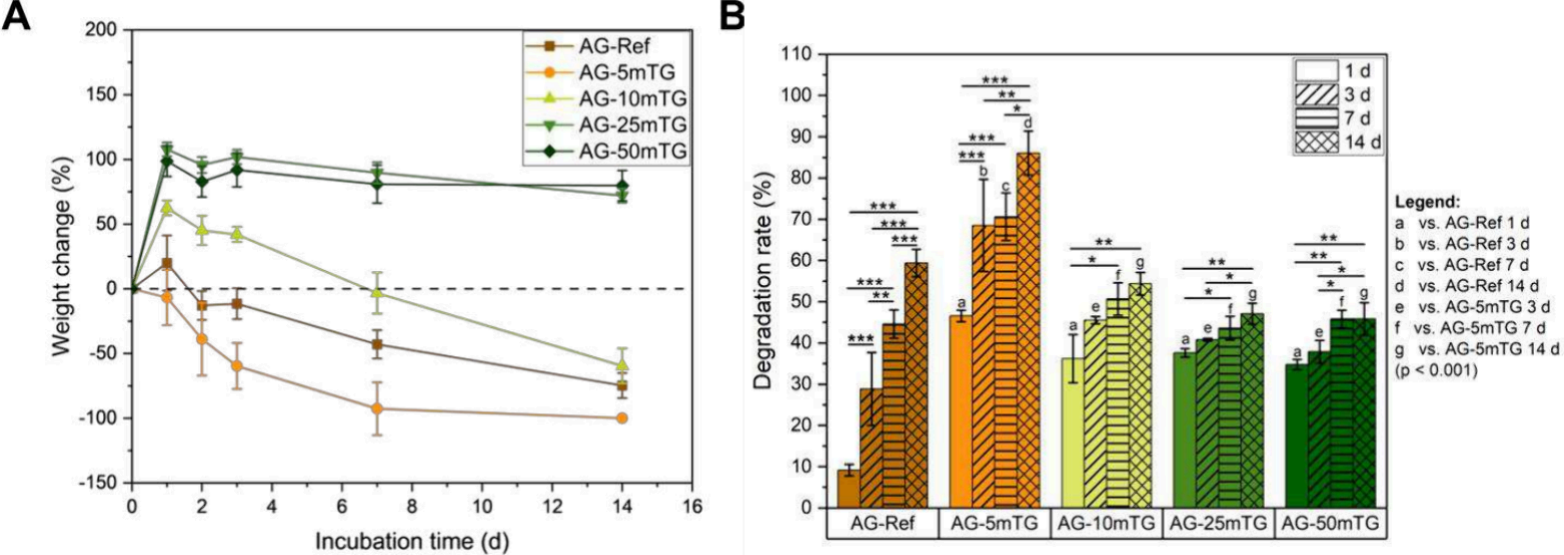
Weight change of *in situ* cross-linked ADA-GEL dependent on mTG concentration up to 14 days of incubation (*n* = 6) (A) and degradation rate based on solid mass (*n* = 3) (B).

The mechanical properties of as-prepared hydrogel films were investigated by compression testing. Figure 3A depicts the effective compressive modulus, which increased with mTG concentration for the *in situ* cross-linking approach. This trend for *in situ* cross-linked hydrogels can be attributed to an enhanced extent of cross-linking, which reduces pore size and increases matrix density, thereby improving mechanical rigidity.^11^ Furthermore, it is important to mention that the determined values are comparable to those of the reference samples, which represents an advantage of the *in situ* cross-linking approach since it allows for a significant reduction of mTG consumption while maintaining similar mechanical properties. The determined compressive modulus of all compositions is comparable to published results by Distler et al.,^6^ reporting approximately 25 kPa for post cross-linked ADA-GEL hydrogels.^6^ Moreover, when comparing the modulus of the tested hydrogel compositions with the mechanical properties of native cartilage tissue, all tested compositions fall within the lower range of the superficial zone of native articular cartilage, ranging from 0.02 to 1.16 MPa.^12^ Furthermore, previous studies on hydrogels for CTE applications reported similar moduli, which supported a native-like morphology of primary human articular chondrocytes and further induced the production of glycosaminoglycans and collagen type II.^13^ As swelling affects the mechanical properties, the effective compressive modulus was further determined after 24 h of incubation at 37 °C (Figure S2). It became evident that the modulus dropped for all compositions with less pronounced decreases for higher mTG concentrations, indicating stronger cross-linking. The drop in modulus can be explained by the onset of degradation and the release of uncross-linked hydrogel components. Additionally, the stress–relaxation behavior of all compositions was investigated since it is known that a faster stress–relaxation is related to an enhanced cartilage matrix formation.^14^ Figure 3B displays the stress–relaxation of all hydrogels. It can be observed that *in situ* cross-linked hydrogels showed a faster relaxation than post cross-linked ADA-GEL. Exemplarily, the normalized compressive stress of AG-Ref was determined to be 0.6 ± 0.02 and decreased to 0.4 ± 0.03 for AG-50mTG after a holding time of 300 s (Figure 3C). The faster relaxation of *in situ* cross-linked hydrogels further became evident with the determined relaxation time, which was defined as the time at which 25% of the initial stress dissipated (Figure 3D). This observation can be explained by the fact that post cross-linked samples may relax stress more slowly due to their heterogeneous cross-linking, preventing efficient stress dissipation across the hydrogel network. In contrast, *in situ* cross-linked samples show a more homogeneous and cohesive network structure, allowing for a more effective rearrangement and stress–relaxation. This behavior is characteristic for viscoelastic and homogeneously cross-linked systems due to the lack of stiff structural domains. Furthermore, a study by Zhao et al.^15^ compared the stress–relaxation behavior of ionically and covalently cross-linked alginate-based hydrogels reporting a faster stress–relaxation of ionically cross-linked hydrogels, which is due to the breakage and reformation of ionic cross-links when exposed to stress compared to covalent cross-linked hydrogels, which show stress–relaxation mainly due to the migration of water.^15^ This explanation could be applied to the *in situ* cross-linked hydrogels presented in this study, which show improved ionic cross-linking due to the presence of CaCl_2_ inside the hydrogel matrix.

**Figure 3:**
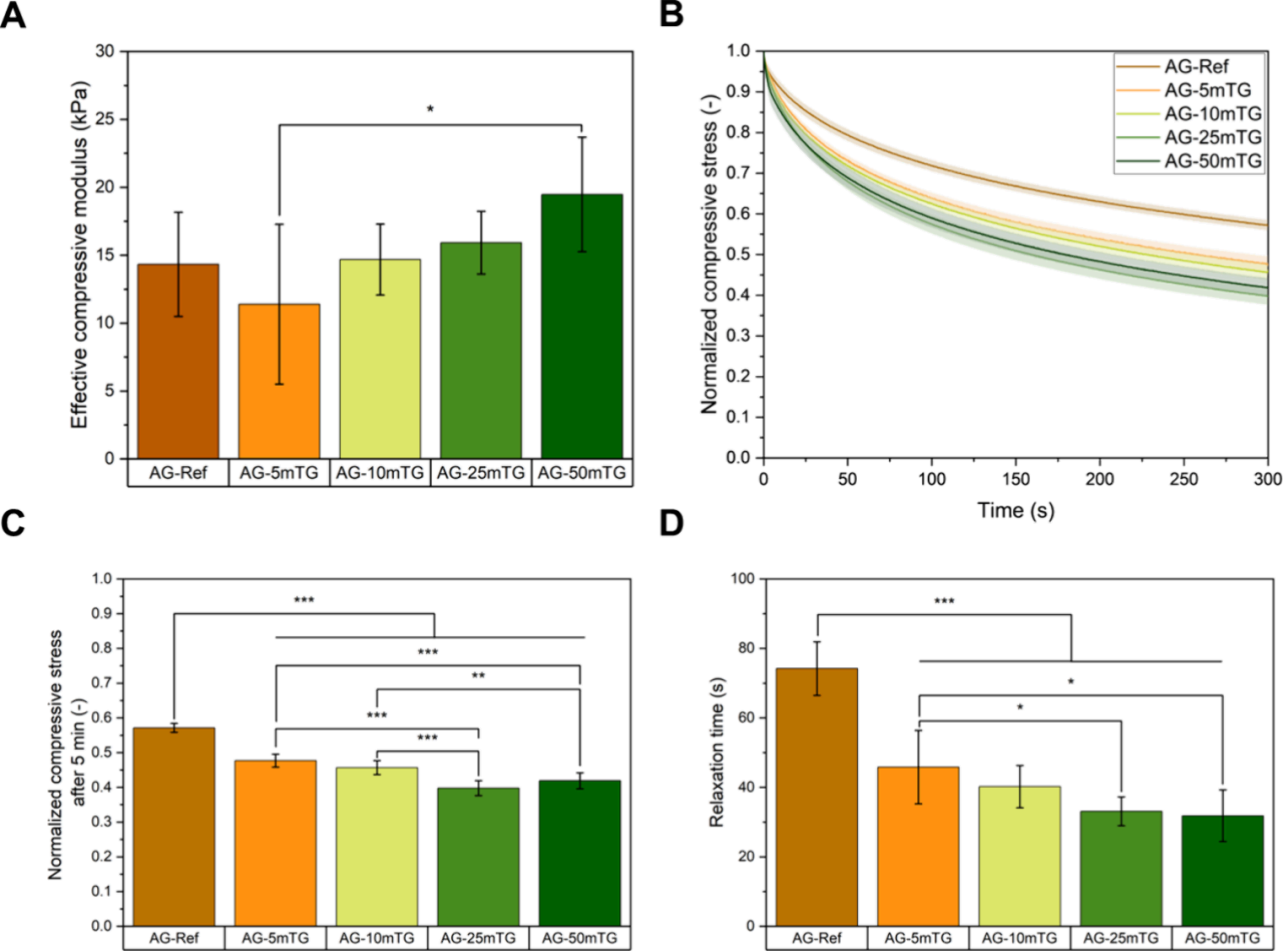
Effective compressive modulus of as-prepared hydrogel films (*n* = 6) (A) and their stress–relaxation behavior (B) (*n* = 6). Normalized compressive stress after 5 min of holding time (*n* = 6) (C) and relaxation time defined as the time at which 25% of the initial stress dissipated (*n* = 6) (D).

Figure 4A displays the mean extrusion force for all investigated compositions, showing that the extrusion force increased steadily until reaching a stable plateau, consistent with the shear-thinning behavior typical of natural hydrogels.^16^ A steady-state extrusion force was established within 20 s for all samples with AG-Ref exhibiting the highest extrusion force and deviation. In contrast, *in situ* cross-linked hydrogel inks demonstrated significantly lower extrusion forces with reduced force fluctuation (Figure 4B). Less fluctuation of the extrusion force indicates greater matrix homogeneity, suggesting a more uniform distribution of cross-links for *in situ* cross-linked samples as expected. Additionally, all compositions exhibited extrusion forces below 6 N, which is far below the clinically acceptable value for injectability of 30 N.^17^ Furthermore, the force deviation remained below 0.5 N, indicating high matrix homogeneity.^18,19^

**Figure 4:**
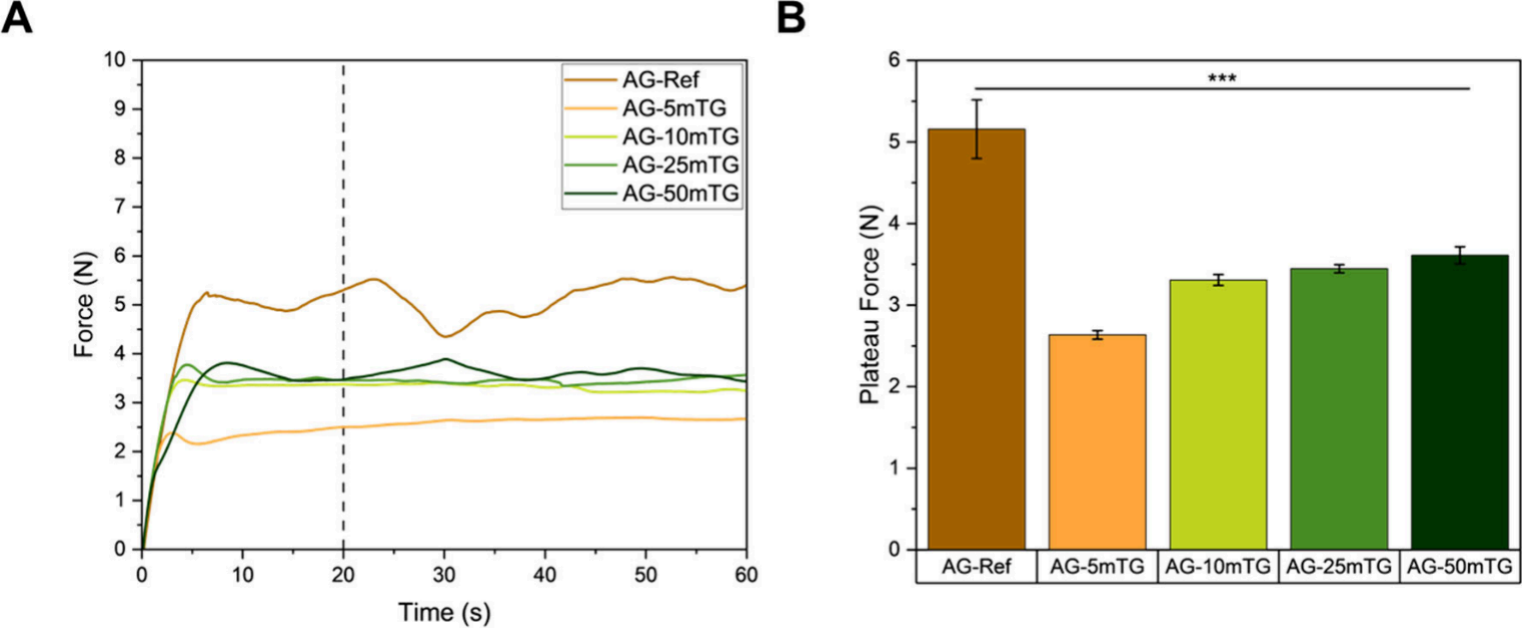
(A) Mean extrusion force over time of ADA-GEL hydrogels with different mTG concentrations (*n* = 3). (B) Plateau force of the hydrogels (*n* =3).

The cytocompatibility of *in situ* cross-linked hydrogels was investigated by encapsulating chondrogenic ATDC5 cells into the hydrogels (Figure 5). Herein, due to the insufficient stability of AG-5mTG and AG-10mTG over the incubation time, we only investigated the cell–material interactions of higher mTG concentrations and the post-cross-linked reference samples. The WST-8 assay (Figure 5A) showed an increased absorbance for ATDC5 cells encapsulated in *in situ* cross-linked hydrogels with significant differences after 7 days compared to the reference ADA-GEL. Furthermore, living cells could be observed for all tested compositions after 7 days based on fluorescence microscopy images of Calcein-DAPI-stained cells (Figure 5B). Moreover, it was observed that the chondrocytes showed a native-like cell morphology, indicating that the hydrogels prevented the dedifferentiation of encapsulated chondrocytes into a fibroblastic phenotype.^20^ The cytocompatibility of ADA-GEL-based hydrogels with various cell types is well described in the literature.^5,7,21^ However, the cell–material interactions of ATDC5 cells with ADA-GEL-based hydrogels have only been studied by cell seeding on a 2D sample; thus, the ability to investigate the cell–material interactions in a 3D environment similar to the native ECM was lacking and is now reported for the first time.^6,21^

**Figure 5:**
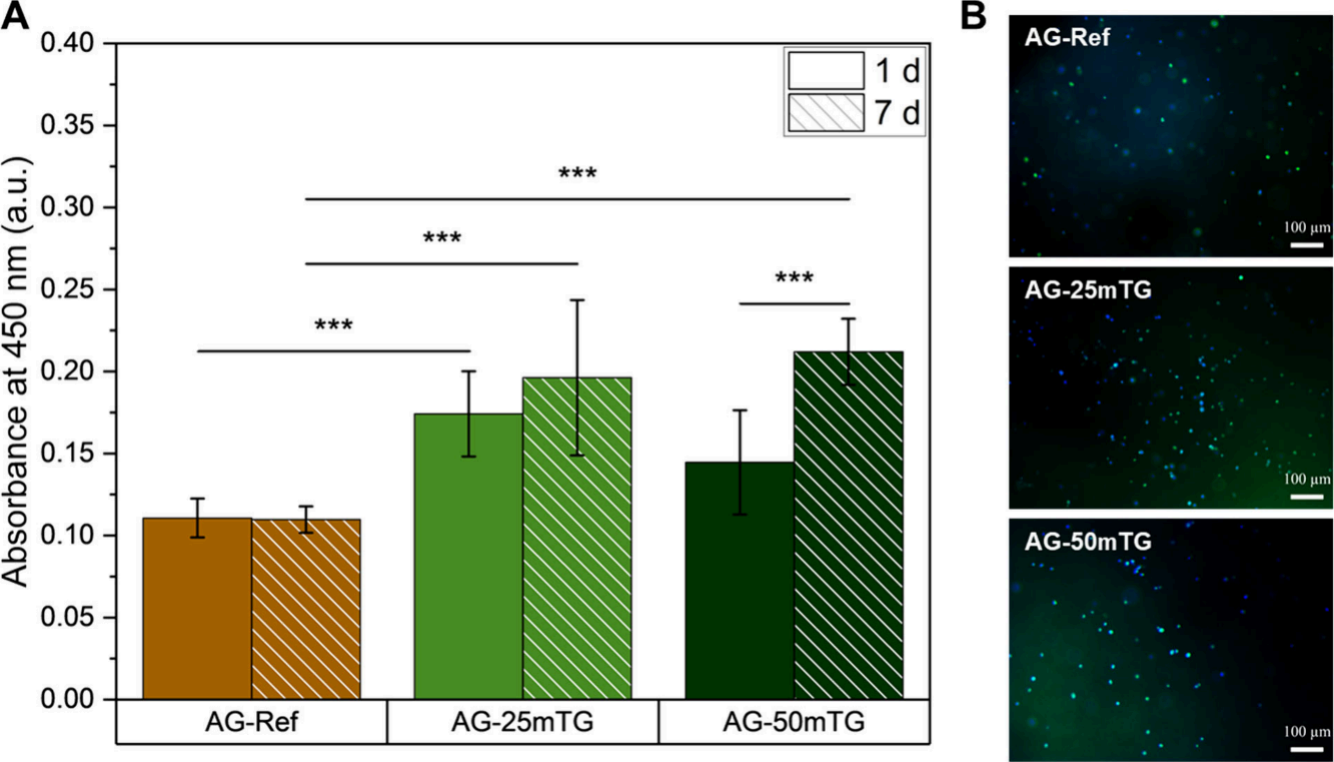
Investigation of cytocompatibility of hydrogels with encapsulated ATDC5 cells by the WST-8 assay (*n* = 3 biological samples) (A) and Calcein-DAPI staining of encapsulated cells after 7 days of incubation (B). Scale bar = 100 μm.

In summary, oxidized alginate-gelatin-based hydrogels were produced by ionic and enzymatic cross-linking employing two different approaches, which were compared in terms of mechanical properties, swelling and degradation behavior, injectability, and cytocompatibility with chondrogenic ATDC5 cells. It was shown that injectable hydrogels could be fabricated using an *in situ* cross-linking approach, allowing for tailorable hydrogel properties. More importantly, similar mechanical properties and improved degradation resistance were achieved compared to the post-cross-linked reference, while the needed concentration of microbial transglutaminase as the enzymatic cross-linker was significantly reduced. Moreover, the *in situ* cross-linking strategy showed promising cell–material interactions with encapsulated ATDC5 cells. Consequently, the proposed *in situ* cross-linking approach represents a facile platform for injectable hydrogels to be used in cartilage tissue repair approaches, allowing for the encapsulation and thus delivery of chondrocytes to the cartilage defect. For future investigations, we envision the use of a double-syringe delivery system, which has the potential to facilitate the application of *in situ* cross-linked ADA-GEL hydrogels in a clinical setting.

## Supplementary Material



## Notes

Complete materials and methods along with a supporting table for a literature overview on *in situ* cross-linked alginate-gelatin hydrogels and supporting figures on the degradation of hydrogels in the presence of collagenase type II as well as the effective compressive modulus of as-prepared hydrogels and after 24 h of swelling (PDF)
