# Supplementary material for: Injectable in Situ Cross-linked Oxidized Alginate-Gelatin-Based Hydrogels for Cartilage Tissue Engineering
Source: ACS Biomater Sci Eng. 2026 Feb 18;12(3):1440–5. doi: 10.1021/acsbiomaterials.5c01832 (PMC12976994; doi:10.1021/acsbiomaterials.5c01832)
Supplement: Supplementary file 1 [file ab5c01832_si_001.pdf]

Supporting Information

Injectable *In Situ* Crosslinked Oxidized Alginate-Gelatin-Based Hydrogels for Cartilage Tissue Engineering

Lisa Schöbel[1,a], Juri Artes[1,a], Markus Lorke[1], and Aldo R. Boccaccini[1,*]

[1] Institute of Biomaterials, Department of Materials Science and Engineering, Friedrich Alexander-University Erlangen-Nuremberg, 91056 Erlangen, Germany

Experimental Section

1. Materials

Alginate (VIVAPHARM®Alginate, PH176) was obtained from JRS PHARMA GmbH & Co. KG, Germany. Microbial transglutaminase (ACTIVA WM) was obtained from Ajinomoto Co.,Inc., Japan. Dulbecco's Phosphate Buffered Saline (DPBS), Hank's Balanced Salt solution (HBSS), Dulbecco's modified Eagle's medium (DMEM, 4.5 g/L D-Glucose, Invitrogen), DMEM/HAMS F-12 (Invitrogen), bovine calf serum (BCS), penicillin/streptomycin (PS, Invitrogen), Calcein AM and DAPI were purchased from Thermo Fisher Scientific Inc., Germany. All other chemicals were procured from Sigma-Aldrich, Germany, if not stated otherwise.

2. Preparation of hydrogel precursor solutions

Alginate was oxidized to alginate dialdehyde (ADA) with an oxidation degree of approximately 19 %, following the protocol described by Karakaya et al.[3] Briefly, 10 g of alginate was dispersed in 50 mL ethanol followed by the addition of an aqueous sodium periodate solution (9.375 mmol, 50 mL). The resulting mixture was stirred for 6 h under dark conditions followed by the addition of 10 mL ethylene glycol to quench the reaction while stirring for additional 30 min. Subsequently, the stirring was stopped and the mixture was left undisturbed to allow for the sedimentation of the reaction product. Finally, the oxidized alginate was transferred into dialysis tubes and dialyzed against 16 L of ultra pure water for 4 days with daily water changes. The reaction product after dialysis was collected, frozen at -21 °C over night and freeze dried (Christ Alpha 1-4 LSCplus, Germany). The resulting ADA was dissolved in DPBS to prepare a 7.941 % (w/v) ADA solution. The gelatin solution (15 % (w/v)) was prepared by dissolving gelatin powder (from porcine skin, gel strength 300 g Bloom, Type A) in either ultra pure water (for reference samples, GEL-Ref) or

0.1 M $CaCl_2$ (ionic GEL) in a water bath at 80 °C for 3 hours under constant stirring as reported by Abroug et al.[2] Microbial transglutaminase solutions were prepared at concentrations of 5 %, 10 %, 25 % and 50 % (w/v) by dissolving mTG powder in DPBS.

3. Preparation of hydrogel films

In order to produce *in situ* crosslinked hydrogel films, the prepared ADA and respective mTG solution were mixed in a beaker under stirring at 37 °C for 3 min. Afterwards, 1 mL of the ADA-mTG hydrogel precursor was transferred into a 2 mL syringe (syringe A). Simultaneously, 1 mL of the ionic gelatin solution was loaded into a second 2 mL syringe (syringe B). The air was squeezed out of both syringes and the syringes were connected with a LuerLock adapter. The components were mixed for 20 transfer cycles, where one cycle corresponds to the complete transfer of the material, yielding an ADA-GEL hydrogel with a final concentration of 3.75 %/7.5 % (w/v). Finally, an 18 G needle was attached to the syringe, and 0.2 mL hydrogel precursor solution was ejected into the cavity of a silicone mold (diameter of 10 mm) to produce a hydrogel film. Subsequently, the molds were transferred to a 37 °C incubator and incubated for their respective gelation time depending on the mTG concentration (see table S1). The gelation time is defined as the needed incubation time to achieve a hydrogel with suitable gel properties for 3D printing and was determined by a bottle-flip test (data not shown). The reference hydrogel samples (AG-Ref) were produced by a post crosslinking approach proposed by Distler et al.[1] using a 0.1 M $CaCl_2$ solution supplemented with 5 % (w/v) mTG.

Table S1: Overview of nomenclature of produced hydrogel specimens, the respective hydrogel volumes, their gelation time, and temperature

Label	Volume of ADA (mL)	Volume of mTG (µL)	Volume of GEL (mL)	Gelation time (min)	Gelation temperature
AG-Ref	0.972	28.0	1.0	30	RT
AG-5	0.972	28.0	1.0	265	37 °C
AG-10	0.972	28.0	1.0	240	37 °C
AG-25	0.972	28.0	1.0	120	37 °C
AG-50	0.972	28.0	1.0	60	37 °C

4. Mechanical Characterization

The effective compressive modulus of as-prepared hydrogel films was tested using the Instron 5967 universal testing machine (Instron, Germany) with a 100 N loading cell. Prior to each measurement, the diameter and height of each specimen were measured using a digital caliper. The compression rate was set to 1 mm/min and the maximum deformation to 20 % strain. The effective compressive modulus was determined from the slope of the stress-strain curve between 5 % and 10 % strain. In addition, stress relaxation experiments were conducted by compressing the hydrogel specimen to 20 % of strain with the subsequent holding of force for 300 s. All tests were performed using six replicates (n = 6).

5. Swelling and Degradation behavior

The swelling behavior was assessed by monitoring the changes in wet weight during incubation under cell culture conditions (37 °C, 5 % CO_2, 95 % humidity). The samples were incubated in DMEM supplemented with 1 % (v/v) PS and weighed periodically over an incubation period up to 14 days. To mimic cell culture conditions, while minimizing the accumulation of degradation by-products, the medium was replaced three times a week. Prior to the start of the experiment, the specimens were transferred to pre-weighed cell strainers, and the sample's initial weight was determined after fabrication (w_0). In the following, the samples were immersed in the incubation medium and after 1, 2, 3 and 6 hours and 1, 2, 3, 7, and 14 days, the inserts were removed from the medium and briefly dried with autoclaved tissue paper before being weighed at the respective time point (w_t). Finally, the weight change was determined using the following formula:

$$Weight\ change\ (\%) = \frac{w_t - w_0}{w_0} \cdot 100\ \%$$

All tests were performed using six replicates (n = 6).

Additionally to the swelling in DMEM, the biodegradability of the hydrogels in presence of collagenase type II was investigated. This investigation was performed as described above except that the DMEM was supplemented with 1 mg/mL collagenase type II. The samples were weighed at specific time points and the weight change was calculated using the earlier presented formula. The biodegradation tests in presence of collagenase type II were performed using three replicates (n = 3).

The degradation rate was investigated by weighing hydrogel samples before and after the incubation in DMEM using their dry weight. The samples were produced as described above and immediately freeze dried. Firstly, the samples were weighed to determine their initial weight (w_0). Subsequently, the samples were re-swollen in HBSS for 1 h at 37 °C in a shaking incubator followed by disinfection in 70 % (v/v) ethanol for 1 h. After the disinfection time had elapsed, the samples were briefly washed with HBSS for 5 min before being immersed in DMEM. At the specific time points, the samples were removed from the medium, briefly washed with ultra pure water and freeze dried. After freeze drying the samples were re-weighed to determine their solid mass after incubation (w_t). The degradation rate was calculated by the following equation:

$$Degradation\,rate\ (\%) = \frac{w_0 - w_t}{w_0} \cdot 100\ \%$$

The degradation rate was performed using four replicates (n = 3) per composition.

6. Injection Force

Injection force measurements were performed using the Instron 5967 universal testing machine (Instron, Germany) equipped with a 100 N load cell. A custom syringe fixture was designed following a recent publication by Falcone et al.[4] to vertically position a 2 mL syringe. The extrusion process was conducted at a constant displacement speed of 5 mm/s for a duration of 60 s using an 18 G needle (840 µm inner diameter). The injection force was measured using n = 3 replicates per composition.

7. Cell-material interactions using the chondrogenic cell line ATDC5

7.1 Cell encapsulation

The cell-material interactions were investigated by encapsulating chondrogenic ATDC5 cells into hydrogels. The cells were cultured in maintenance medium (DMEM/HAMS F-12) supplemented with 5 % (v/v) fetal bovine serum, 1 % (v/v) PS, 30 nM sodium selenite, and 10 µg/mL human transferrin in an incubator at 37 °C, 5 % CO_2, 95 % humidity. On the day of encapsulation, the cells were detached from the surface of T-75 flasks using trypsin and counted by the trypan blue exclusion method using a Neubauer cell counting chamber. For the post crosslinking samples, 10^6 cells/mL hydrogel were mixed with the ADA-GEL solution for 5 min at 37 °C. In case of the *in situ* crosslinked hydrogels, 10^6 cells/mL hydrogel were added to the ADA-mTG precursor solution

and subsequently mixed with ionic GEL using the syringe-mixing method as described in section 3. Finally, the cell-laden hydrogels were added to a 48 well-plate (0.2 mL per well) and crosslinked as described in section 3 followed by covering the samples with medium and incubation up to 7 days.

7.2 Metabolic activity a*ssay*

The metabolic activity of encapsulated ATDC5 cells was analyzed using a water-soluble tetrazolium salt-based WST-8 metabolic assay (Cell Counting Kit-8, Sigma-Aldrich) using three replicates per composition (n = 3). After the respective incubation period, the medium was removed from the wells and replaced with a 3 % (v/v) WST-8 solution. After an incubation time of 3 hours at 37 °C, 5 % CO_2, 95 % humidity, technical triplicates of 100 µL for each sample were pipetted into a 96-well plate and the absorbance at 450 nm was measured using a microplate reader (FLUOstar Omega, BMG Labtech, Germany) resulting in n = 9 measurements per composition. The absorbance values were used as determined and not corrected against the background signal of acellular hydrogels.

7.3 Calcein AM-DAPI staining

After performing the WST-8 assay, a Calcein AM-DAPI staining was performed. The Calcein-AM staining solution was prepared at a concentration of 4 µL Calcein AM per mL HBSS under dark and sterile conditions. After removing the WST-8 solution from the wells, the Calcein AM staining solution was added and the samples were incubated for 45 min at 37 °C. Subsequently, the Calcein AM solution was removed and the cells were fixed using a 4 % (v/v) formaldehyde solution for 15 min at room temperature. Afterwards, a DAPI solution containing 1 µL DAPI per mL HBSS solution was added and the staining was performed for 30 minutes at room temperature in the dark. Finally, the solution was removed and HBSS was added for sample preservation. The samples were stored at 4 °C in the dark until examination by fluorescence microscopy (AXIO Observer.D1, Carl Zeiss Microscopy, Germany).

8. Statistical Analysis

Statistical analyses were performed by one-way analysis of variance (ANOVA) using Origin (Origin 2024b, OriginLab, USA). All data are presented as means with ± standard deviation. Different levels of significance between different material compositions were evaluated using

Holm-Bonferroni analysis. Significance levels are shown as: $p < 0.05$ = *, $p < 0.01$ = ** and $p < 0.001$ = ***.

Supporting Content

Table S2: Literature overview on *in situ* crosslinked and/or injectable hydrogels for tissue engineering applications

Material	Type of *in situ* CL	Post CL performed	Injectability Characterization	Ref
ADA-GEL with calcium-silicate particles	Schiff's Base and release of divalent ions from inorganic filler particles	Yes	Not reported	(1)
Alginate with $CaCO_3$ and D-(+)-gluconic acid δ-lactone (GDL)	Internal ionic gelation by controlled release of Ca^{2+} after reaction between $CaCO_3$ and GDL	Yes	Not reported	(2)
Alginate-GEL-Xanthan Gum-Mesoporous bioactive Glass (MBGN)	In situ ionic CL of Alginate by Ca^{2+} ions released from MBGNs	Yes	Injection force ranging from 17 – 20 N (21 G)	(3)
ADA-GEL	Schiff's Base in presence of Borax	No	Not reported	(4)
Hydrazide-modified Poly(L-glutamic acid)-ADA	Schiff's Base	No	Qualitatively reported by imaging	(5)
ADA-PEG-GEL and ADA-PEG-carboxymethyl chitosan	Schiff's Base	No	Acceptable injection forces below 10 N (21 G)	(6)
GEL-mTG	Enzymatic crosslinking	No	Not reported	(7)
GEL-oxidized pectin	Schiff's Base	No	Not reported	(8)
Phenolic hydroxyl group modified GEL	Peroxidase-catalyzed enzyme reaction	No	Not reported	(9)

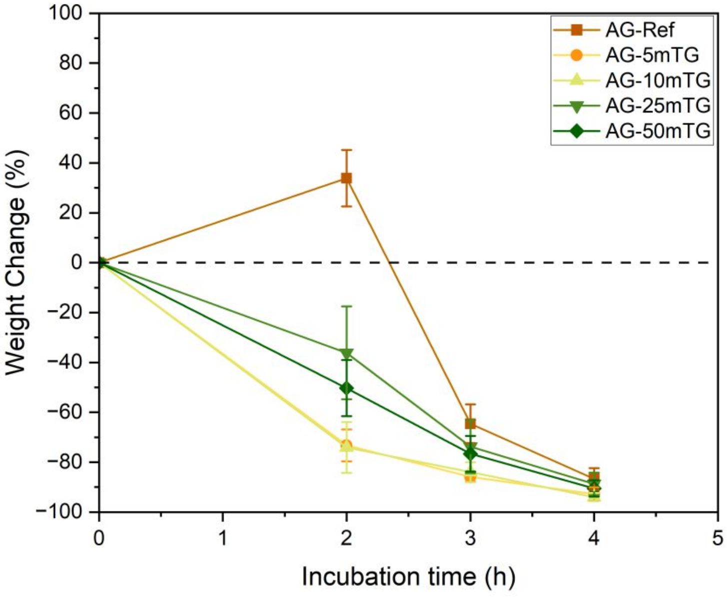

Figure S1: Degradation of as-prepared hydrogels in collagenase type II supplemented DMEM at 37 °C (n = 3).

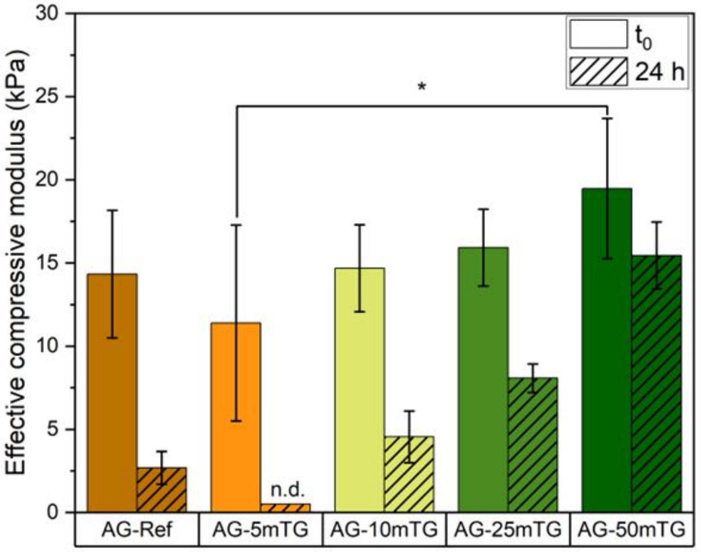

Figure S2: Determined effective compressive modulus of as-prepared hydrogel specimen and samples after 24 h of swelling in DMEM at 37 °C (n = 6). Due to the fragility of *in situ* crosslinked ADA-GEL hydrogel specimens with 5 % mTG, this group could not be tested after 24 h of incubation.

References

(1) Heid, S., Becker, K., Byun, J., Biermann, I., Neščáková, Z., Zhu, H., Groll, J., & Boccaccini, A. R. (2022). Bioprinting with bioactive alginate dialdehyde-gelatin (ADA-GEL) composite bioinks: Time-dependent in-situ crosslinking via addition of calcium-silicate particles tunes in vitro stability of 3D bioprinted constructs. Bioprinting, 26. https://doi.org/10.1016/j.bprint.2022.e00200

(2) Hazur, J., Detsch, R., Karakaya, E., Kaschta, J., Teßmar, J., Schneidereit, D., Friedrich, O., Schubert, D. W., & Boccaccini, A. R. (2020). Improving alginate printability for biofabrication: establishment of a universal and homogeneous pre-crosslinking technique. *Biofabrication*, *12*(4). https://doi.org/10.1088/1758-5090/ab98e5

(3) Damian-Buda, A. I., Lorke, M., Boccaccini, A. R., & Unalan, I. (2025). Novel Antioxidant and Antibacterial Injectable Hydrogels Incorporating Clove Oil-Loaded Mesoporous Bioactive Glass Nanoparticles: A Promising Strategy for Enhanced Bone Regeneration. *Macromolecular Bioscience*, *25*(11). https://doi.org/10.1002/mabi.202500252

(4) Balakrishnan, B., & Jayakrishnan, A. (2005). Self-cross-linking biopolymers as injectable in situ forming biodegradable scaffolds. *Biomaterials*, *26*(18), 3941–3951. https://doi.org/10.1016/j.biomaterials.2004.10.005

(5) Yan, S., Wang, T., Feng, L., Zhu, J., Zhang, K., Chen, X., Cui, L., & Yin, J. (2014). Injectable in situ self-cross-linking hydrogels based on poly(l -glutamic acid) and alginate for cartilage tissue engineering. *Biomacromolecules*, *15*(12), 4495–4508. https://doi.org/10.1021/bm501313t

(6) Naghizadeh, Z., Karkhaneh, A., & Khojasteh, A. (2018). Self-crosslinking effect of chitosan and gelatin on alginate based hydrogels: Injectable in situ forming scaffolds. *Materials Science and Engineering C*, *89*, 256–264. https://doi.org/10.1016/j.msec.2018.04.018

(7) Alarake, N. Z., Frohberg, P., Groth, T., & Pietzsch, M. (2017). Mechanical properties and biocompatibility of in situ enzymatically cross-linked gelatin hydrogels. *International Journal of Artificial Organs*, *40*(4), 159–168. https://doi.org/10.5301/ijao.5000553

(8) Gupta, B., Tummalapalli, M., Deopura, B. L., & Alam, M. S. (2014). Preparation and characterization of in-situ crosslinked pectin-gelatin hydrogels. *Carbohydrate Polymers*, *106*(1), 312–318. https://doi.org/10.1016/j.carbpol.2014.02.019

(9) Sakai, S., Hirose, K., Taguchi, K., Ogushi, Y., & Kawakami, K. (2009). An injectable, in situ enzymatically gellable, gelatin derivative for drug delivery and tissue engineering. *Biomaterials*, *30*(20), 3371–3377. https://doi.org/10.1016/j.biomaterials.2009.03.030
